# Antimicrobial Resistance in the Food Chain: Trends, Mechanisms, Pathways, and Possible Regulation Strategies

**DOI:** 10.3390/foods11192966

**Published:** 2022-09-22

**Authors:** Mrinal Samtiya, Karl R. Matthews, Tejpal Dhewa, Anil Kumar Puniya

**Affiliations:** 1 Department of Nutrition Biology, Central University of Haryana, Mahendergarh 123029, India; 2 Department of Food Science, Rutgers University, New Brunswick, NJ 08901, USA; 3 Dairy Microbiology Division, ICAR-National Dairy Research Institute, Karnal 132001, India

**Keywords:** antimicrobial resistance/AMR, food chain, foodborne infection, pathogens, food safety, pathways of antimicrobial resistance, regulatory guidelines

## Abstract

Antimicrobial resistance (AMR) remains of major interest for different types of food stakeholders since it can negatively impact human health on a global scale. Antimicrobial-resistant bacteria and/or antimicrobial resistance genes (transfer in pathogenic bacteria) may contaminate food at any stage, from the field to retail. Research demonstrates that antimicrobial-resistant bacterial infection(s) occur more frequently in low- and middle-income countries (LMICs) than in developed countries. Worldwide, foodborne pathogens are a primary cause of morbidity and mortality. The spread of pathogenic bacteria from food to consumers may occur by direct or indirect routes. Therefore, an array of approaches both at the national and international level to control the spread of foodborne pathogens and promote food safety and security are essential. Zoonotic microbes can spread through the environment, animals, humans, and the food chain. Antimicrobial drugs are used globally to treat infections in humans and animals and prophylactically in production agriculture. Research highlights that foods may become contaminated with AMR bacteria (AMRB) during the continuum from the farm to processing to retail to the consumer. To mitigate the risk of AMRB in humans, it is crucial to control antibiotic use throughout food production, both for animal and crop agriculture. The main inferences of this review are (1) routes by which AMRB enters the food chain during crop and animal production and other modes, (2) prevention and control steps for AMRB, and (3) impact on human health if AMR is not addressed globally. A thorough perspective is presented on the gaps in current systems for surveillance of antimicrobial use in food production and/ or AMR in the food chain.

## 1. Introduction

Antibiotic therapy is one of the dominant strategies of contemporary medicine used to fight bacterial infections. From the 1930s to the 1960s most antibiotics available today were developed; the period is considered the ‘golden era’ of antibiotics [1]. Failure to develop or discover new antibiotics and indiscriminate use of existing antibiotics without proper guidelines are contributing factors that have led to antibiotic resistance [2]. AMR has become a general threat to the prevention and management of bacterial infections [3]. Swann was one of the first to sound the alarm about the problems linked to indiscriminate use of antibiotics, suggesting that the enormous amount of antibiotics used without following norms could be unsafe for human health [4].

Several decades later, with disregard for warnings, data shows, for example, from 2014 to 2016, nearly a million people died due to antimicrobial-resistant infections, and the outlook is bleak, with premature mortality of nearly 300 million people by 2050 [5]. The relative ease of accessibility and low cost of antibiotics for treating infections have improved human health and life expectancy. Perhaps an unintended consequence of ample availability and low cost is off-label and unregulated widespread use and development of AMR. AMR occurs when microbes (i.e., bacteria, fungi, and viruses) alter their physiology or genetic makeup following frequent contact with antimicrobial agents (i.e., antibiotics). For example, ‘Superbugs’ are bacteria resistant to multiple antimicrobials [6]. Factors contributing to the ‘global resistome’ or AMR include excessive overuse of antibiotics, an international movement of people and food, poor hygiene, and the release of non-digested antibiotics into the environment [7]. The impact on humans is exemplified by foodborne illnesses associated with antimicrobial-resistant strains of *Salmonella enterica* serovar Typhimurium of pork and poultry origin [8,9]. Antibiotics are often used prophylactically in food-producing animals (i.e., cattle, chickens, and pigs); it is predicted that by 2030 such use will increase by nearly 67% globally [10]. The abundant use of antibiotics raises concerns about increased AMR in microbes. The World Health Organization (WHO) reports that although 50 newer antibiotics and 10 biologicals are under evaluation, they target only 32 WHO-priority pathogens, and for the remaining pathogens, no targeted antimicrobials have been developed [11]. To provide better treatments, the WHO and ‘Drugs for Neglected Diseases Initiative’ by 2025 have formed a non-profit organization called ‘Global Antibiotic Research and Development Partnership’ to develop new antibiotics that challenge antimicrobial-resistant organisms [11]. In LMICs, the prospect of AMR leads to increased illness and mortality that may be linked to limited patient presentation, reduced accessibility to diagnostics, and poor access to second-line antibiotics [12]. Research establishes a link between the indiscriminate overuse of antibiotics in agricultural production and the increase in AMR of human infections [8,9,13,14,15,16,17,18]. AMRB can spread to humans indirectly through ingesting contaminated food and interacting with animals harboring AMRB and biologicals, including blood, urine, feces, saliva, and semen [19]. The use of antimicrobials, biocides, and heavy metals in the food and agriculture sector impacts the emergence of AMRB. AMR genes may also contaminate food; for example, a previous study showed that plasmid-borne ampicillin-resistant genes transfer into *Escherichia coli* K12 from *Salmonella* Typhimurium DT104 in ground meat and inoculated milk [20].

The Food and Agriculture Organization (FAO), World Organization for Animal Health (OIE), and WHO recommended the ‘One Health’ approach supporting healthy animals, healthy people, and healthy environments [21,22]. LMICs are far behind in the resources available for implementing the ‘One Health’ initiative and reducing the spread of AMRB. AMR has spread rapidly through the globalization of the food supply, increased population in urban areas, and international travel [23].

## 2. Trends in AMR

The worldwide spread of antibiotic-resistant microbes substantially raises the risk to public health [24]. The concerns surrounding AMR are exacerbated by the lack of discovery of new antibiotics. Even when antibiotics are used properly, a few cells may survive and transfer resistance characteristics, creating bacteria that may ultimately become multi-antibiotic resistant [23,25]. One of the foremost reasons associated with the spread of antibiotic resistance is the widespread overuse of antibiotics linked with the lack of enforcement of regulations [26]. Major health problems are related to the global AMR crisis, with an estimated 0.7 million deaths globally linked to AMR. This figure may increase to 10 million by 2050 [27]. These AMR threats are growing daily due to the occurrence of resistivity in the bacterial strains for antibiotics, so this figure may be more in the future [28].

AMR in bacteria occurs through spontaneous mutation(s) and the transfer of genetic material (e.g., transposons, plasmids). The paucity in developing new antibiotics limits the number of effective antibiotics against multi-antibiotic-resistant bacteria and permits the increase in the spread of AMRB [29]. In 2014, the WHO stated that in its six reporting regions > 25% of *Streptococcus pneumoniae* were resistant to penicillin, and five out of the six regions > 50% of *Escherichia coli* were resistant to third generation cephalosporins. AMR surveillance data is limited in many regions globally, although available data suggest that in Africa nearly 100% of bacteria are resistant to β-lactam antibiotics [12]. Antibiotic-resistant bacteria are associated with urinary tract infections, tuberculosis, sepsis, gonorrhea, and foodborne illnesses. Common medical procedures, including surgery, organ transplantation, neonatal care, diabetes, and chemotherapy, present an increased human health risk without effective antibiotics [6,30].

Research confirms that in at least ten countries, third-generation cephalosporins fail to treat *Neisseria gonorrhoeae*. Similarly, carbapenem-resistant *Klebsiella pneumoniae* has spread worldwide [31]. The efficacy of commonly used antimicrobials has been reduced because of AMR, for example, *Acinetobacter*, *Pseudomonas*, *E. coli*, *K. pneumoniae*, *Salmonella enterica*, *Staphylococcus aureus*, and *S. pneumoniae* that are often linked to nosocomial infections [31,32].

AMRB adversely impacts LMICs since those countries often lack the resources to mitigate the spread of AMRB [33]. Exposure to 1000 colony-forming units (CFU) of cephalosporin-resistant *E. coli* (CREC) through consumption of contaminated chicken meat was estimated at the rate of 1.5%; the greatest concern was actually through cross-contamination in the kitchen during a meal [8]. Aquaculture practices that include the indiscriminate use of antimicrobial agents are linked to an increase of AMRB isolated from farm-raised fish [34,35]. Antimicrobial-resistant microbes are seemingly ubiquitous and can spread to new environmental niches while transferring resistance to other bacteria [36,37]. Presently, no single approach is adequate to mitigate the occurrence and spread of AMR. Without a synchronized and multi-sectoral ‘One Health’ approach, the world may revert to the pre-antibiotic era [6,7].

## 3. Mechanisms in AMR

Different mechanisms are employed by microbes against antimicrobial agents: degradation of antibiotics/antibacterial agents by enzymes, modification of antibiotic targets, altering cell wall permeability, and activation of alternate pathways [38]. AMR is recognized as an international threat and is an exquisite example of the rapid adaptation of microbes to a new bionetwork [39]. A common mechanism of resistance in bacteria is the enzymatic degradation of antimicrobial agents. Resistance to aminoglycosides is primarily facilitated by enzymatic degradation using acetyltransferases, nucleotidyltransferases, and phosphotransferases [40]. An example of a naturally arising resistance mechanism influencing bacteria’s survival is resistance to β-lactam antibiotics; β-lactamases have existed for thousands of years [41]. A study on breastfed babies found that on the first day of breastfeeding 14.3% of isolated Enterobacteriaceae were extended-spectrum β-lactamase (ESβL) positive, increasing to 41·5% by the 60th day in the breast-fed infants [42].

Saprophytic microbes produce antimicrobial molecules that impede the growth of adjacent microbes, thereby acting as a survival strategy. The antimicrobial molecules produced even at sublethal levels affect bacterial physiology and adaptive microbial evolution and possibly act as signaling molecules that influence the expression of microbial and host genes [43]. Resistance can be achieved by modification of the targeted antibiotic, where the antibiotic fails to bind and exert a negative effect on the bacterial cell. Mutations associated gyrase and topoisomerase genes are examples of this type of mechanism that are targets of the quinolone and fluoroquinolone antibiotics [44]. Gut microbiota of a pre-Columbian Andean mummy (980–1170 AD) harbored β-lactam, quinolones, fosfomycin, chloramphenicol, macrolide, sulfa, tetracycline, aminoglycoside, and vancomycin resistance genes [45]. Resistance to fluoroquinolones has been widely documented, with 10–40% of clinical *E. coli* resistant to fluoroquinolones [23,46]. Fluoroquinolone resistance mechanisms used by bacteria include modification of target (i.e., DNA-gyrase), increased efflux (removal of the drug outside by the cell), inactivation of fluoroquinolone (by aminoglycoside N-acetyltransferase), and targeting DNA-binding proteins [47].

Antimicrobial exposure enhances selective pressure through the survival and proliferation of bacteria having intrinsic resistance or newly attained resistance (e.g., mutations) [41]. The widespread use of antibiotics exerts selective pressure on commensal microbiota and pathogens, increasing the likelihood of recovery of AMRB from patients [48]. The alteration of cell-wall permeability and regulation of efflux systems are mechanisms the cell may employ to protect itself from certain types of antibiotics. For example, decreased sensitivity to tetracycline is associated with genes related to efflux systems [49]. Intrinsic AMR systems are found in different genera and species of bacteria. Concerns are infections linked to opportunistic antibiotic-resistant pathogens such as *Clostridium difficile* in humans or *Trueperella* (*Arcanobacterium*) *pyogenes* in bovines that can ultimately result in death [50,51].

AMR genes can be acquired through vertical gene transfer (VGT; parent to progeny) and horizontal gene transfer (HGT; transmission of cell-to-cell genetic material). VGT and HGT may happen simultaneously. HGT results in a population of microbes having enhanced resistance profiles through the acquisition of new resistant genes and mechanisms [52]. HGT can occur using pili (conjugation), bacteriophage (transduction), or assimilation of extracellular DNA (transformation) [5]. HGT permits interspecies transmission and is a prominent driving force in the spread of AMR [23,25].

## 4. Pathways of AMR in the Food Chain

**Figure 1:**
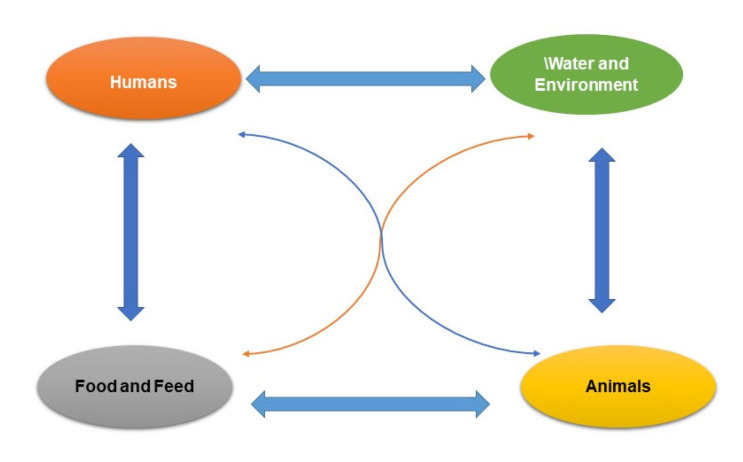
Depicting the potential routes of AMR in the food chain.

Food is an excellent vehicle for spreading AMR spoilage and pathogenic bacteria. Notably, an increase in AMRB in food would have a negative effect on human health. The degree to which AMR is spread worldwide through the food supply may not be fully appreciated [53]. AMRB may enter the food supply at any time during the farm-to-fork continuum. AMRB that contaminates products at the farm level is likely to remain on raw and undercooked foods going to the consumer [54,55,56,57,58,59]. The routes of exposure to AMRB are indirect through consumption of food and direct through contact with infected animals or biological constituents (i.e., blood, urine, feces, saliva, semen) [19]. The potential pathways of AMR in the food chain are shown in Figure 1.

### 4.1. Spread through Foods of Animal Origin

Foods of animal origin are a primary source of AMRB in the food chain [53]. Central to this are domesticated livestock and the meat derived from animals that are harboring AMRB. The spread of AMR *Salmonella* is generally linked to contaminated poultry meat, eggs, pork, and beef. AMR *Salmonella* has also been linked to turkey [60,61]. In addition to AMRB, AMR genes have been reported in food products derived from poultry, swine, goats, cattle, and sheep [62,63,64]. Seafoods grown in aquaculture systems and farms are designated as “Hotspots of AMR” due to the more significant genetic exchange, which makes seafood more susceptible to gaining resistivity. AMR in food derived from aquaculture could reduce antibacterial effectiveness in humans. Aquaculture also made indirect transmission of resistant genes from aquatic environments (bacteria) to the pathogens related to humans [65,66] possible. Several studies demonstrate that antibiotic-resistant microbes and AMR genes found in humans are present in animals that have not been in contact with humans. This suggests the transfer of AMR to humans through the consumption of contaminated food and improper food handling [52,67]. Poultry is one of the most prominent vehicles for the transmission of AMR *Campylobacter* [53,68]. Quinolone-resistant *E. coli* have been recovered from cattle and the surrounding farm environment [69]. In the study, nearly 60 % of the *E. coli* isolates were recovered from calves and about 28% from the fecal matter of cows. Results showed that there was substantial variability in the recovery of *E. coli* farm samples. The prevalence of antibiotic-resistant *Salmonella*, *Campylobacter*, and *E. coli* associated with veterinary samples is being tracked [70]. Large differences in the incidences of antimicrobial-resistant *E. coli* associated with veal, beef, and dairy products have been reported by Catry et al. [71].

### 4.2. Spread Associated with Non-Animal Origin Foods

The surveillance data of AMRB associated with foods of non-animal origin are quite limited. Outbreak data between 2007 and 2011 indicate that foods of non-animal origin were linked with 10% of foodborne pathogen outbreaks [72]. Certain key non-animal origin food categories contribute to the AMR problem, including but not limited to *Salmonella-*contaminated leafy greens, stem vegetables, tomatoes, and melons. Contamination of legumes and grain with AMR *E. coli* contributes to the spread of AMR [72].

### 4.3. Environment and Water Spread of AMRB

AMRB isolated from foods is found in the animal production environment [73,74,75]. Research demonstrates the spread of *Staphylococcus aureus* at great distances through attachment to dust particles that travel through the air [76]. Microbes in the atmosphere frequently form large bunches of dust particles. Dispersed microbes contaminate the environment: soil, water, vegetation, and eventually animals/humans [77,78]. *Coliforms* normally associated with feces are widely dispersed in the environment, particularly in areas adjacent to livestock production operations [79]. Methicillin-resistant *S. aureus* (MRSA) has been recovered from environmental samples [80]. Friese et al. [81] reported that 85.2% of air samples collected from swine-housing environments were positive for livestock-associated MRSA. Dust was identified as a significant vehicle for the spread of MRSA through the air. Drug-resistant *S. aureus* isolated from samples collected inside and outside pig-housing units were likely spread by dust [82]. Dust particles may have been involved in the spread of extended-spectrum β-lactamases-*Enterobacteriaceae* [83]. Water is a crucial vehicle for the spread of extracellular mobile genetic elements associated with AMRB-resistant organisms [84,85].

Drinking water contaminated with animal or human feces containing AMRB can be a vehicle for spreading AMRB and antibiotic residues. The gastrointestinal tract of humans and animals can become colonized with AMRB due to consuming water contaminated with AMRB [86]. Water acts as a very effective vehicle for spreading AMRB, genetic elements, and antibiotic residues with the potential to contaminate the environment, crops, and livestock and ultimately result in human illness.

Nonpathogenic bacteria can also be a source of antibiotic resistance genes [85]. Hölzel et al. [87] predicted that AMRB could spread in the food chain through the use of untreated human and animal manure as fertilizer. Consequently, the spread of AMRB, resistant genes, and antibiotics associated with human wastewater or animal manure cannot be overlooked. Vital et al. [88] indicated that multidrug-resistant bacteria were isolated from irrigated water, soil, and vegetable samples collected from urban farms, suggesting that water serves as a vehicle for the extensive spread of AMRB across different environments.

### 4.4. Spread-Associated Food Handlers and Food Contact Workers

Food handlers are frequently associated with the spread of AMRB resulting from poor hygienic practices or cross-contamination from handling contaminated food. Food handlers may harbor AMR *E. coli*, although limited information supports contamination of food by such individuals [89,90]. Non-thermal technologies (high-pressure, ionizing radiation, ultraviolet radiation, and pulsed electric field) for food processing and preservation have been developed to improve the microbial safety of food while retaining nutritional and sensory qualities. Since these technologies damage cell membranes, the potential exists for the release and transfer of genetic material associated with AMR to environments the food contacts [53]. McMahon et al. [91] reported that sub-lethal preservation methods of food using heat, acid, and salt could considerably change the phenotypic characteristics of AMRB (including *E*. *coli*, *S. typhimurium*, and *S. aureus*). When used at a sub-lethal concentration(s), certain biocides can precipitate the development of AMR and/or decreased sensitivity to antimicrobial agents. The single exposure of a strain of *S. typhimurium* to a biocide resulted in multidrug resistance in the strain [92]. Other probable routes of introducing AMR in foods include bacteria added to food during fermentation or as probiotics. Studies have demonstrated that AMRB has occasionally been isolated from fermented foods containing probiotics [93,94]. Speculatively, another potential route for the spread of AMR is through genetically modified plants. During genetic modification, AMR marker genes are used to enable the identification of transformed cells. The resistance genes may potentially transfer to commensal bacteria associated with plants, soil, and animals [95,96].

## 5. Correlation between AMR and Usage of Antimicrobials

Antibiotics are the most prescribed drugs; approximately half are not required [97]. The driving force of AMR is the inappropriate use of antimicrobial drugs [3,30]. The use of antimicrobials during the production of foods such as meat, milk, and products derived from these is of major concern since antimicrobial residues may remain in the foods intended for human consumption. Considerable shifts in etiological agents of bacteremia and the associated antimicrobial susceptibility profiles of those agents have been noted. In clinical settings, the rapid administration of a suitable antimicrobial drug is required to treat, for example, bacteremia, but with the emergence of AMR, treatment choices are becoming limited [98]. The development of AMR is a common evolutionary progression for microbes, but this is accelerated due to selective pressure caused by inappropriate use or overuse of antimicrobials [99]. There is a compelling link between AMR and the excessive use of antimicrobials [100,101,102,103].

In humans and animals, a range of pathogens (*E. coli*, *S. enterica*, *Campylobacter* spp., and *S. aureus*) may cause illness. The evidence supports that in the last couple of decades in LMICs, consumption of antimicrobials has increased [104,105]. Patterns have evolved with respect to the overuse of antimicrobials agents; prescribing to comply with a patient’s request even when the infection is non-bacterial, prescribing an antibiotic that is considered a ‘last resort’ treatment, use of improper dosage or administration, and failure to adhere to a treatment regimen. Comprehensive data are lacking on the efficacy of antimicrobial use, but in Organization for Economic Co-operation and Development countries, nearly half of antimicrobial drugs are considered ineffective for heatlhcare [106].

The extensive use of antibiotics as growth-promoting agents for food animals and aquaculture exacerbates the increase in AMRB [107]. The appearance of AMRB in the food chain is an issue linked to the extensive use of antibiotics in aquaculture, livestock, and crop production [52,107]; the ease of the spread of AMRB and antibiotic-resistant genes at every phase of the food chain [107,108]; and human disease [19,21]. The administration of antibiotics prophylactically and as growth promotants to animals increases the probability that trace amounts of antibiotics may contaminate food and feed. Animals receiving antibiotics as growth promotants often carry antibiotic-resistant bacteria, including MRSA, antibiotic-resistant *Campylobacter* spp., and extended-spectrum β-lactamase (ESBL) producing Enterobacteriaceae [62,109,110,111]. Overuse of antibiotics leads to resistant strains that may contaminate the food supply [23,25]. Enhanced surveillance, implementation, and adherence to the guidelines and regulations on the use of antibiotics in food production and human medicine are essential [112].

## 6. Status of AMR in the Food Chain

Antimicrobials include antibiotics and related semi-synthetic or synthetic agents that demonstrate antimicrobial efficiency and discriminating toxicity [113]. The emergence of AMR limits the therapeutic possibilities of an antimicrobial, both for clinicians and veterinarians, impacting human and animal health. The WHO issued a report indicating that the antibiotics being developed against pathogens that present the greatest risk to human health are insufficient to control the expanding AMR problems [31]. If not properly regulated, AMR’s impact on mortality is alarming, with mortality increasing from 700,000 to 10 million yearly by 2050 [114].

From 1994 to 2000, AMR contagions were 6, 17, and 22% for the USA, Kuwait, and China, respectively. By 2050, it is predicted that AMR will drop the gross domestic product by 2–3.5%, with a decrease in livestock of 3–8%, substantially impacting the global economy [27]. Cases of MRSA bacteremia decreased by 81% between 2007 and 2013; however, total carbapenemase-producing Enterobacteriaceae increased tenfold between 2009 and 2014 [115]. AMR emergence results in about 25,000 mortalities in the US annually, and the number may vary depending on types of resistance and associated infections [116]. Patient data from 2001 to 2015 in France indicate that multidrug-resistant bacteria (>2 antibiotics) were uncommon (37 of 27,681 patients), with four deaths of which three were attributed to other reasons [117].

India published approximately 2152 studies on AMR of which 1040 (48.3%) were associated with humans, 70 (3.3%) with animals, 90 (4.2%) with the environment, and 11 (0.5%) linked to ‘One Health’. The remaining publications included novel agents, diagnostics, editorials, and miscellaneous subject areas related to AMR [118]. Estimation of the AMR problem in LMICs is based on extrapolation; for example, neonatal sepsis attributed to drug-resistant infection was estimated at 214,500 in the year 2012 based on an estimation of all neonatal deaths attributable to severe infection and drug-resistant infection in first-line management [119].

AMR frequently includes ‘one world’ reflecting the ‘One Health’ approach and a universal problem that links food systems and travel [120]. The ‘One Health’ concept encompasses problems that have inter-relatedness between human health, animal health, food, and the environment and raises common efforts on the part of regulatory agencies to address those challenges [121]. This is exemplified by a research paper that included an animated map displaying the worldwide spread of the ‘New Delhi metallo-β-lactamase 1’ or ‘NDM-1’ resistance gene [122]. Another example is the ‘mobilized colistin resistance 1’ or ‘MCR-1’ gene. The MCR-1 gene was initially isolated from pigs and humans in China [123]. Colistin resistance was <1%, but colistin-resistant *K. pneumoniae* was linked to high mortality of up to 70% [124]. Researchers reported the occurrence and frequency of antibiotic-resistant *S. aureus* in 80 samples of meat and chicken. The study was conducted on two swine farms 45% of workers were colonized with the same MRSA strain that was isolated from swine. In the study, *S. aureus* isolated in 67.5% of the samples were resistant to methicillin, and 87.5% were resistant to bacitracin [125]. A report from India on AMR indicates that more than 70% of *E. coli*, *Klebsiella pneumoniae*, and *Acinetobacter baumannii* isolates and almost 50% of *Pseudomonas aeruginosa* were resistant to third generation cephalosporins and fluoroquinolones [118]. Additional studies indicated that among Gram-positive bacteria, 42.6% of *S. aureus* were methicillin-resistant, and 10.5% of *Enterococcus faecium* were vancomycin-resistant. For *Salmonella* Typhi and *Shigella* species, 28 and 82% were resistant to ciprofloxacin, 0.6 and 12% to ceftriaxone, and 2.3 and 80% to co-trimoxazole, respectively. *Vibrio cholera* showed resistivity rates against tetracycline that varied from 17 to 75% [126]. More than 2.8 million antibiotic-resistant infections occur annually in the US, with more than 35,000 deaths. In 2017, 223,900 cases of *Clostridium difficile* occurred, and at least 12,800 people died [127]. Table 1 summarizes various AMR bacteria and their presence in food samples.

## 7. Microbes Displaying Resistance

The CDC is concerned about the occurrence of community-acquired AMRB. The incidence and development of previously non-antibiotic-resistant microbes remain among the most significant concerns. A CDC report listed 18 AMRB and fungi; some of those microbes are presented in the following section [127]. Table 2 shows the selected microbes that developed drug resistivity, as well as the approved antibiotic drugs for the treatment of infection associated with those microbes.

### 7.1. Carbapenem-Resistant Acinetobacter (CRA)

CRA causes pneumonia, urinary tract infections, and wound infections. From a food perspective, *Acinetobacter* is linked to the spoilage of meats and vegetables. *Acinetobacter* may contain mobile genetic elements that are effortlessly transmitted among bacteria. Some strains produce a carbapenemase enzyme that protects the cell from damage. *Acinetobacter* is an emerging risk to hospitalized patients, as it is a fomite contaminating common medical equipment in clinical settings. *Acinetobacter baumannii* is of considerable concern since it cannot be treated with existing antibiotics. In 2017, CRA infected nearly 8500 hospitalized patients, resulting in approximately 700 deaths in the US [127].

### 7.2. Carbapenem-Resistant Enterobacteriaceae (CRE)

*Enterobacteriaceae* include spoilage and foodborne pathogens. They may be isolated from fresh vegetables, soil, and irrigation water. CRE is an imminent concern to patients in healthcare settings. Patients that require devices such as catheters and may take antibiotics for a long duration are at maximum risk of infections with CRE. CRE also harbors mobile genetic materials that can be spread easily between other microbes. Around 30% of CRE carry a mobile genetic component encoding for enzyme production that targets carbapenem antibiotics eliminating these drugs as a treatment option. Some of the most pathologically related *Enterobacteriaceae* are *E. coli*, *Klebsiella pneumoniae*, and *Enterobacter* species which are common infectious agents of the intra-abdominal region and urinary tract infections [127,138].

### 7.3. Drug-Resistant Campylobacter

*Campylobacter* is a major cause of foodborne illness and is associated with raw poultry and unpasteurized milk. *Campylobacter* commonly causes diarrhea (bloody), fever, abdominal pains, and sometimes sequelae such as irritable bowel syndrome, Guillain-Barre syndrome, and arthritis. Around 29% of all infections are associated with strains that have reduced sensitivity to fluoroquinolones or macrolides (azithromycin), antibiotics used to treat severe *Campylobacter* infections. *Campylobacter* spp. are a prominent cause of diarrheal infections and deaths (*n* = 109,700) in 2010 [127,139].

### 7.4. ESBL Producing Enterobacteriaceae

Foods such as seed sprouts (alfalfa, radish) may be favorable for *Klebsiella*. *Klebsiella* is not considered a foodborne pathogen, but foodborne isolates may be ESBL-positive. ESBLs are enzymes that target antibiotics such as penicillins and cephalosporins. CTX-M, a specific ESBL enzyme, emerged in bacteria in the US and spread internationally. The genes encoding the CTX-M enzyme can be transferred to different *Enterobacteriaceae* species. The combination of CTX-M and ST131 enhances resistance and may spread in combination [127]. *E. coli* carrying CTX-M genes are common and considered primary contributors to spreading resistance genes across species and/or geographic regions [140,141]. In tropical and subtropical regions, 25 to 50% of infections are linked to ESBL-E, and in the healthy population, the carriage is 20 to 40% in regions endemic to ESBL-E [140,142].

### 7.5. Vancomycin-Resistant Enterococcus (VRE)

CDC’s ‘National Healthcare Safety Network’ specified that central line-associated bloodstream infections are most often caused by Vancomycin-resistant *Enterococcus faecium*. Over 70% of *E. faecium* are vancomycin-resistant, the antibiotic of choice for treating *E. faecium* infections. The development of vancomycin resistance may be linked to the egregious use of vancomycin to treat MRSA and *C. difficile* infections [143]. *Enterococcus* species may be used in starter cultures, so there is a concern that VRE may be spread by food. However, VRE is not considered a foodborne pathogen.

### 7.6. Methicillin Resistant S. aureus

*S. aureus* is of concern in healthcare facilities as well as in the community. MRSA was initially discovered in 1968 in association with nosocomial infections and has since become community-acquired [144]. *S. aureus* infections can be challenging to treat since the pathogen may resist methicillin and several other vital antibiotics. *S. aureus* is a foodborne pathogen that causes food poisoning due to toxin production in food.

### 7.7. Drug-Resistant Non-Typhoidal Salmonella

Non-typhoidal *Salmonella* may be responsible for diarrhea, fever, and abdominal pains. People may acquire *Salmonella* infections by eating contaminated foods or after contact with the feces of infected people or animals. Antibiotics used to treat patients with *Salmonella* infections include ciprofloxacin, azithromycin, and ceftriaxone. Infections caused by resistant strains of *Salmonella* may be more severe and result in higher rates of hospitalization. In 2018, it was reported that *Salmonella enterica* serovar Infantis was associated with 25% of infections. The majority of infected people had no history of travel but had eaten chicken [127]. Almost all AMR *Salmonella* infections are foodborne and linked to the consumption of contaminated pork, turkey, and beef [145]. In 2017, 59,066 deaths were due to non-typhoidal *Salmonella* infection [146]. International traveling has been acknowledged as a contributing risk for *Salmonella* infection [147].

## 8. Strategies to Regulate AMR

The foodborne infections associated with AMR are foremost among key public health concerns. Infections caused by AMRB substantially increase the morbidity and mortality rates, especially in the developing world, while in developed nations, the therapeutic costs increase due to these infections [148]. The WHO created a ‘Strategic and Technical Advisory Group’ on AMR and endorsed that the WHO should be a primary party in forming the action plan. The FAO launched its Plan for Antimicrobial Resistivity to support WHO’s global action plan in food and agricultural regions [21]. The One Health approach was proposed by international bodies to control AMR risks, forming an association between WHO, FAO, and OIE as a ‘tripartite alliance’. WHO, also initiated a plan to stabilize this worldwide issue in association with tripartite partners and issued a ‘Global Action Plan’ on AMR [22]. Emphasizing the possible strategies to regulate concerns of AMR, the five strategic objectives of WHO’s Global Action Plan are shown in Table 3 and as follows:

### 8.1. Increase Awareness of AMR via Active Communication, Education, and Training

Steps should be taken simultaneously to increase the awareness of AMR and support behavioral transformation through community communication programs that consider diverse audiences in human and animal health and agricultural practices [21]. ‘World Antibiotic Awareness Week’, as a part of its Global Action Plan was started by the ‘WHO’. WHO also issued guidance for a skilled program for health workers’ learning and training, recognizing discrepancies in the quality and analysis of initiatives to strengthen the education and training on AMR of healthcare workers [149]. Awareness programs are supported by mass media, and social media repeated messaging regarding issues related to AMR that may decrease antibiotic usage and AMR rates [148]. A program started by the joint partnerships Wellcome Trust/DBT India Alliance called ‘superheroes against superbugs’ was started in India. Its main aim was to include school students as partners to engage creatively with the public on AMR [150]. The intention behind these efforts was to change human behavior, social beliefs, and lack of education on the use of antibiotics and AMR [151]. The OIE provides guiding principles on primary veterinary education, emphasizing a planned profession with well-trained experts [152].

### 8.2. Support Knowledge through Observation

Resolving the issues linked to AMR requires the implementation of suitable activities supported by a strong rationale for their cost-effectiveness as well as widespread benefits. Organizations, including intergovernmental, professional, and non-governmental, along with industry and academia each have significant roles to play in enhancing and transforming such knowledge into practice [22]. The USA ‘National Antimicrobial Resistance Monitoring System’, ‘Danish Integrated Antimicrobial Resistance Monitoring and Research Programme’, and ‘Antimicrobial Resistance and Antibiotic Usage in Animals in the Netherlands’ are well-known programs formed by developed nations to collect data related to AMR and the food supply [153,154,155]. Indeed, to control AMR it is important to realize how resistance increases and spreads. Understanding how AMR moves in the network of humans, animals, food, water, and the environment is relevant for developing new tools, guidelines, and laws to regulate AMR [22]. The ‘Colombian Integrated Program for Antimicrobial Resistance Surveillance’ (COIPARS) is a model for surveillance of foodborne infections by the ‘International Molecular Subtyping Network’ as well as by the WHOs ‘Global Foodborne Infections Network’ [156]. The WHO ‘Advisory Group on Integrated Surveillance of Antimicrobial Resistance’ used COIPARS methods for the regulation of AMR and the use of antibiotics in food animals [156]. The WHO (2015) indicates that 6 out of 47 and 6 out of 21 countries of the WHO African and Eastern Mediterranean Regions, respectively, have national reference laboratories that evaluate the antibiotic susceptibility of microbes [157]. In developing nations, revision of research policies for food animals, food-derived products, and humans is needed to control the spread of AMR from farm to fork [158].

### 8.3. Decrease the Occurrence of Infectious Diseases through Cleanliness, Hygiene, and Preventive Measures

AMR bacteria arise in clinical settings (e.g., hospitals), the result of seriously ill patients requiring extensive antibiotic therapy needing a range and volume of antibiotics for treatment. To prevent infections, it is necessary to take robust measures to reduce the growth and dispersal of multidrug-resistant bacterial and antimicrobial-resistant infections [22]. A recent UN report emphasizes that 97 out of 158 countries are complying with infection prevention and control programs [159]. According to the WHO, an analysis of country self-assessments indicated about 58% of responding countries reduced spread by implementing actions for sanitation, hygiene, and infection prevention, with around 25% reaching full implementation of measures [160]. Water and sanitation facilities are more accessible for hospitals in urban regions than rural regions [161]. It is difficult to measure the influence of water and sanitation planning on the spread of AMR. At least one study suggests that the spread of AMR is linked to poor sanitation and polluted potable water more than selection pressure related to the substantial use of antibiotics [162].

### 8.4. Regulate the Use of Antimicrobials in Human and Animal Health

Evidence suggests that AMR is more likely to spread due to the substantial, improper use of antimicrobials. Widespread AMR has been linked to inappropriate use of antibiotics, the result of over-prescription and ease of accessibility through Internet sales [22]. In Denmark, avoparcin (growth promoter) was the first antibiotic that was banned (in 1995) followed by virginiamycin in 1998 for use in animals as a growth promotant and an inclusive ban of antibiotics in 2000 [153,163]. There was a significant reduction noted in antibiotic use and AMR after the ban, although it had little effect on pig and poultry yields with a small drop in productivity initially at the time of the ban [164]. Veterinary use guidelines should be compulsory, especially for antibiotics used to treat infectious diseases in animals and humans. It is also essential to develop measures to confirm the judicious use of antibiotics and establish targets for evaluating progress and strategies for utilizing veterinary antibiotics [152,165]. The ‘Global Antimicrobial Resistance Surveillance System’ was launched to support the antimicrobial resistance global action plan. The main objective was to advance the worldwide surveillance of AMR in humans to support the evidence on AMR and to encourage decision making in addition to driving national, regional, and global actions [166]. In 2018, 71 countries were registered in ‘The Global Antimicrobial Resistance Surveillance System’. According to a UN report, only 29 of 106 countries with national surveillance systems are LIMCs [159]. OIE endorsed the strengthening of veterinary legislation and implementation policies to confirm compliance with laws and regulations that support the responsible and judicious use of ‘Veterinary Critically Important Antimicrobial Agents’, ‘Veterinary Highly Important Antimicrobial Agents’, and ‘Veterinary Important Antimicrobial Agents’ [152]. It is therefore essential that every country include stakeholders from different sectors such as government, industry, experts, practitioners, and international bodies to set an achievable and practicable target to decrease antibiotic consumption [27].

### 8.5. Improve Economic Situation for Sustainable and Increased Investment in Novel Drugs, Diagnostic Tools, and Vaccines

An assessment of the economic impacts on health is required to evaluate the broader socioeconomic problem of antimicrobial resistance. Assessments should compare the cost of doing nothing compared to the cost of action. There is a need for investment in the advancement of novel antimicrobial treatments, as well as in analytical tools and vaccines. The shortage in such investment reveals the fears for rapidly occurring AMR, and returns on investment also are limited due to restrictions in use [22]. The financial burden related to humans with existing AMR infections is insignificant compared to the cost of not investing in new drug discoveries. AMR should be a nationwide priority to ensure public attention, confirm investment, and assign resources to contain it positively. The formation of robust partnerships between the public and private sectors is required to develop solutions to the problem. In the food industry, an active political drive is essentially needed to enact sustainable engagement, investment, research, and alternatives to antibiotics [165,167]. To overcome the global challenge of AMR, multi-sectorial and organized efforts should be cohesively identified and research priorities targeted while providing appropriate funding [27,167]. A comprehensive plan is needed to reduce the challenge of AMR in the community and for the development of effective alternatives to the use of antibiotics in production agriculture [164]. Synthetic bacteria with useful functionalities can be used to bio-synthesize novel antibiotics with unique antibacterial activity under artificial and engineered regulatory methods [168].

## 9. Conclusions

Antimicrobial resistivity is affecting the global population, resulting in health and financial losses. The ‘One Health’ concept is supported by the ‘World Organization for Animal Health’ and WHO, under which suitable approaches can be developed and implemented to control AMR. Currently, the major focuses are on antimicrobial residues in food that may occur due to the indiscriminate use of antibiotics in agriculture. Food and foodstuffs can be contaminated with AMRB at any farm-to-table continuum point. Two major steps need to be monitored to overcome or stop the risk of AMRB in the food chain, i.e., antimicrobial use in foods and AMRB originating from agricultural practices. The developed approaches should be policy-based, enforced for all countries, and entirely backed by government regulations. No action taken by a single country will resolve the AMR problems facing the global food supply, but a collective global approach will surely do so.

## Figures and Tables

**Table 1:** Different AMR bacteria in various food samples (plant and animal origin).

AMR Microorganisms	Food Samples	Country/Region	References
*Escherichia coli*, *Listeria monocytogenes*, *Staphylococcus aureus*, and *Salmonella* spp.	Raw bovine milk	Iran	[128]
ESBL producing *E. coli*	Pigs, broiler, fish, etc.	Thailand (Different selected areas)	[129]
*Salmonella*, *L. monocytogenes*, and *S. aureus*	Raw milk, cooked food products, and raw meat	China	[130]
*Campylobacter jejuni*, *C. coli*, *C. iari*	Poultry products	Africa	[131]
*C. jejuni*, *C. coli*	Poultry meat and neck skin	Sri Lanka	[132]
*Escherichia coli*, *Salmonella*, *Vibrio cholerae*, *Staphylococcus*	Meats#br#products,#br#dairy#br#products, etc.	Cuba	[133]
Methicillin-resistant *Staphylococcus aureus* (MRSA)	Pigs	-	[134]
*Aeromonas*	Catfish and eel farms (aquaculture)	Netherlands (southern)	[135]
*Salmonella* species	Animal origin food (such as cream cake, egg sandwich, raw meat, raw milk, etc.)	Ethiopia	[136]
*Bacillus* spp., *Erwinia* spp.,*#br#Ewingella americana. Staphylococcus* spp., *Enterobacter cloacae*, and *Stenotrophomonas maltophilia*	Herbal products (such ginger root, garlic powder, etc.)	-	[137]

**Table 2:** Selected microbes that developed drug resistivity coupled with the release year of antibiotic drugs.

Resistant Identified (Year)	Antibiotic Released (Year)
Amphotericin B-resistant *Candida auris* (2016)	Amphotericin B; 1959
Azithromycin-resistant *N. gonorrhoeae* (2011)	Azithromycin; 1980
Ceftazidime-avibactam-resistant KPC-producing *K. pneumonia* (2015)	Ceftazidime-avibactam; 2015
Ciprofloxacin-resistant *N. gonorrhoeae* (2007)	Ciprofloxacin; 1987
Daptomycin-resistant methicillin-resistant *S. aureus* (2004)	Daptomycin; 2003
Extended-spectrum β-lactamase-producing *E. coli* (1983)	Extended-spectrum cephalosporins; 1980 (Cefotaxime)
Fluconazole-resistant *Candida* (1988)	Fluconazole; 1990 (FDA approved)
*K. pneumoniae* carbapenemase (KPC)-producing *K. pneumonia* (1996)	Imipenem; 1985
Methicillin-resistant *Staphylococcus aureus* (1960)	Methicillin; 1960
Penicillinase-producing *N. gonorrhoeae* (1976)	Penicillin; 1941
Penicillin-resistant *S. aureus* (1942)	
Penicillin-resistant *S. pneumonia* (1967)	
Plasmid-mediated vancomycin-resistant *Enterococcus faecium* (1988)	Vancomycin; 1958
Vancomycin-resistant *S. aureus* (2002)	Vancomycin; 1958

Content source: Centers for Disease Control and Prevention, National Center for Emerging and Zoonotic Infectious Diseases (NCEZID), Division of Healthcare Quality Promotion (DHQP) Available online: https://www.cdc.gov/drugresistance/biggest-threats.html (accessed on 20 November 2020)

**Table 3:** Possible strategies to regulate concerns of AMR.

Possible Strategies	Consequence
Increase awareness	Awareness programs are supported by mass, and social media repeated messaging regarding issues related to AMR, which may decrease antibiotic usage and AMR rates.
Support knowledge through observation	Organizations (govt/non-govt) along with industry and academia can improve the practical knowledge to combat AMR concerns
Cleanliness, hygiene, and preventive measures	Proper hygiene and cleanliness by following necessary guidelines can help to decrease AMR issues.
Regulate the use of antimicrobials	Guidelines should be compulsory, especially for antibiotics used to treat infectious diseases in animals and humans.
Improve economic situation	There is a need for investment in the advancement of novel antimicrobial treatments, analytical tools, and vaccines. Shortage in such investment reveals the trends of continued AMR.

## Data Availability

Not applicable.

## Abbreviations

AMR	Antimicrobial resistance
AMRB	AMR bacteria
LMICs	low- and middle-income countries
GLASS	Global Antimicrobial Resistance Surveillance System
NARMS	National Antimicrobial Resistance Monitoring System
OIE	World Organization for Animal Health
FAO	Food and Agriculture Organization
WHO	World Health Organization
HGT	Horizontal gene transfer
VGT	Vertical gene transfer
MRSA	Methicillin-resistant *S. aureus*
ESβL	Extended spectrum-β-lactamase
MCR-1	Mobilized colistin resistance-1
CRA	Carbapenem-resistant Acinetobacter
CRE	Carbapenem-resistant Enterobacteriaceae
CDC	Centers for Disease Control and Prevention
COIPARS	Colombian Integrated Program for Antimicrobial Resistance Surveillance
